# Enrichment Bayesian design for randomized clinical trials using categorical biomarkers and a binary outcome

**DOI:** 10.1186/s12874-022-01513-z

**Published:** 2022-02-27

**Authors:** Valentin Vinnat, Sylvie Chevret

**Affiliations:** ECSTRRA Team, INSERM U1153,Université de Paris, 1 avenue Claude Vellefaux, Paris, 75010 France

**Keywords:** Bayesian study design, adaptive enrichment design, sensitive subpopulation

## Abstract

**Background:**

Adaptive clinical trials have been increasingly commonly employed to select a potential target population for one trial without conducting trials separately. Such enrichment designs typically consist of two or three stages, where the first stage serves as a screening process for selecting a specific subpopulation.

**Methods:**

We propose a Bayesian design for randomized clinical trials with a binary outcome that focuses on restricting the inclusion to a subset of patients who are likely to benefit the most from the treatment during trial accrual. Several Bayesian measures of efficacy and treatment-by-subset interactions were used to dictate the enrichment, either based on Gail and Simon’s or Millen’s criteria. A simulation study was used to assess the performance of our design. The method is exemplified in a real randomized clinical trial conducted in patients with respiratory failure that failed to show any benefit of high flow oxygen supply compared with standard oxygen.

**Results:**

The use of the enrichment rules allowed the detection of the existence of a treatment-by-subset interaction more rapidly compared with Gail and Simon’s criteria, with decreasing proportions of enrollment in the whole sample, and the proportions of enrichment lower, in the presence of interaction based on Millen’s criteria. In the real dataset, this may have allowed the detection of the potential interest of high flow oxygen in patients with a SOFA neurological score ≥ 1.

**Conclusion:**

Enrichment designs that handle the uncertainty in treatment efficacy by focusing on the target population offer a promising balance for trial efficiency and ease of interpretation.

**Supplementary Information:**

The online version contains supplementary material available at (10.1186/s12874-022-01513-z).

## Background

Phase III trials often require large sample sizes, leading to high costs and delays in clinical decision-making. Moreover, these trials often include heterogeneous populations. On one hand, these populations offer the potential for larger sample sizes in a shorten accrual time. On the other hand, the risk for negative findings due to potential treatment-by-subset interactions exist. This notion was recently exemplified in the COVID-19 pandemic, where an overwhelming number of clinical trials have been registered to test a variety of preventive and therapeutic strategies [1] with negative meta-analysis findings [2]. If negative findings could be explained by variation in health-care resource availability [3], they could also be due to the large inter-individual variations in patient profiles [4]. Rather than enrolling all diseased patients into the trial, one instead may enroll only those whose profile indicates that they could benefit from the treatment, thus targeting a very selective population for whom the test drug likely works.

Adaptive clinical trials have become more common in recent years to allow inclusion of more than one potential target population into one trial without conducting trials separately [5]. Such so-called “enrichment designs” allow the eligibility criteria of the trial to be iteratively updated during the trial, restricting entry to patients likely to benefit from the new treatment. These trials mostly use frequentist approaches [6], raising the issues of repeated statistical tests and lack of power. More recently, some enrichment designs have proposed the use of Bayesian modelling, partitioning the population into separate blocks [7, 8] or using predictive probabilities of response according to the patient profile to allocate patients [9]. This approach is consistent with the growing literature that proposes Bayesian approaches to adaptive clinical trials [10–12].

We placed ourselves in the setting of a randomized clinical trial with 2 parallel arms and a categorical biomarker. Potential treatment-by-subset interactions appear at the core of precision medicine, which is evaluated properly through stratified designs [13] whereby all patients are randomized between the treatment and the control, and the randomization is stratified on the subset status [14]. Bayesian measures of interaction have been previously proposed by Millen [15] and Morita [7], although in a different setting. Morita [7] introduced a subset selection criterion based on the posterior measure of influence of the treatment. Millen [15] additionally introduced a criterion based on the posterior treatment-by-subset interaction. We believe that combining both of these criteria will maximize the probability of identifying the subset, which could benefit the most from the experimental treatment. Therefore, we sequentially assessed treatment-by-subset interactions in a Bayesian framework.

The main objective of this work was to use Bayesian treatment-by-interaction measures to derive an adaptive clinical trial design that evaluates the therapeutic intervention of any targeted therapy and identifies subsets of subjects who respond better (or worse) to the experimental therapy to enrich the enrolled population.

## Motivating trial

In the HIGH multicenter randomized clinical trial, a total of 776 immunocompromised patients admitted to the intensive care unit with hypoxemic acute respiratory failure (ARF) were randomized 1:1 to either continuous high-flow oxygen therapy (n=388) or to standard oxygen (n=388) (The HIGH study registration NCT, NCT02739451. Retrospectively registered on 15 April 2016). The sample size was computed to demonstrate a decrease in the day-28 mortality rate from 30% in the standard oxygen therapy group to 20% in the high-flow oxygen therapy group, demonstrating a relative risk of 0.67.

No evidence of any impact of the initial oxygenation strategy was observed on the 28-day mortality (high-flow oxygen therapy 35.6% vs. standard oxygen 36.1%; P=0.94)[16]. We wondered whether there could be differential treatment effects on sub-populations of varying ages or type of organ dysfunctions as measured by the SOFA sub-scores, focusing on four partitions according to: (i) patient age distinguishing (a) 2 subsets (< 65 versus ≥65 years) according to the mean value or (b) 3 subsets (≤58 versus >58& ≤68 versus ≥68 years) according to the terciles; (ii) neurological disorders (SOFA neurological <1 versus ≥1); and (iii) oxygenation ratio PaO2/FiO2 (<100 versus ≥100). Note that prevalence of the smallest subset varied from 10% up to 44%.

**Fig. 1:**
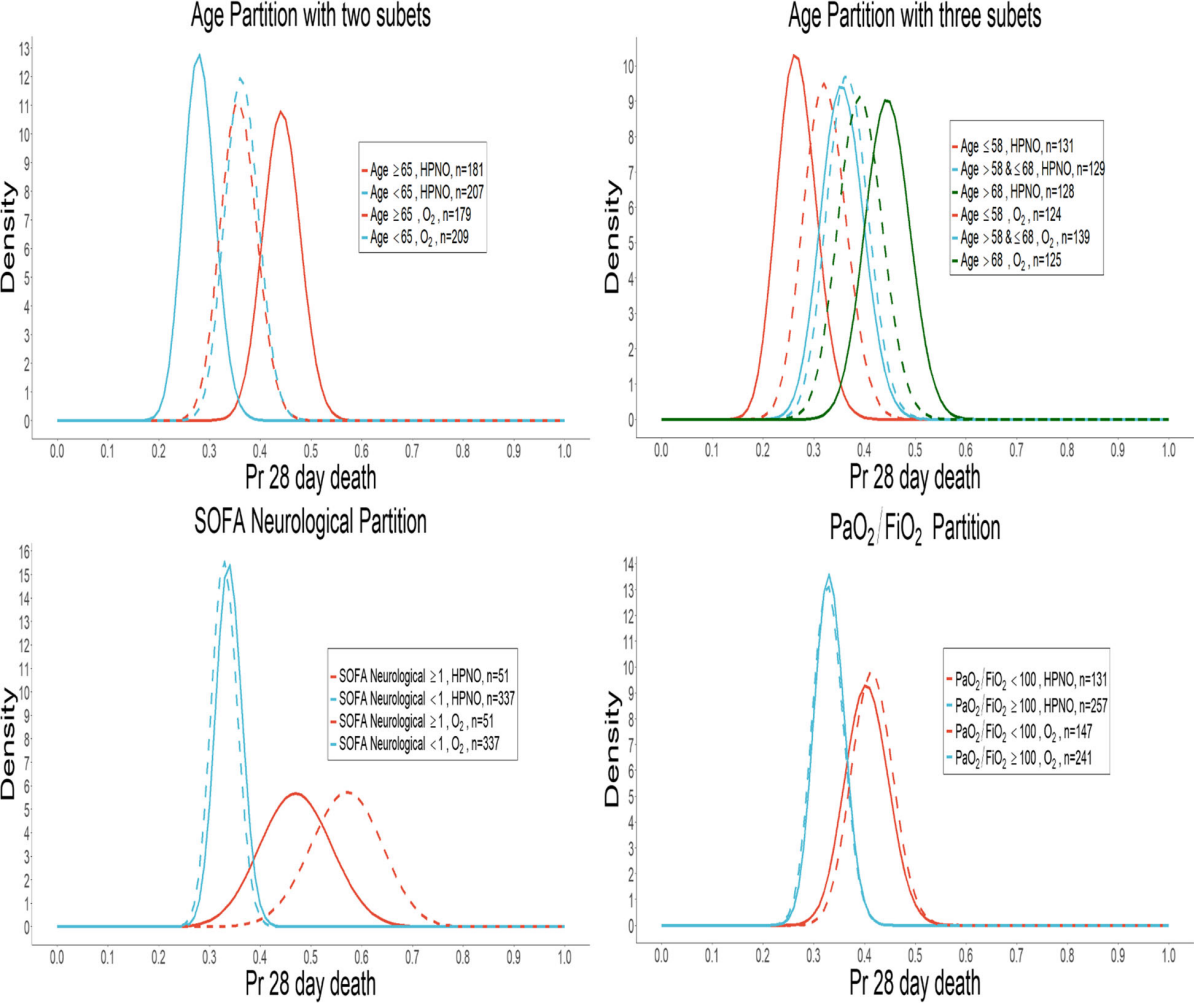
HIGH Trial: Posterior probabilities of probability of death in both randomized groups, according to patient subsets. In each randomized group in each subset, a Beta-binomial model was used to model the probability of death, where a non-informative Beta(1,1) prior was actualized in a Beta (1+*r*,1+*n*− *r*) posterior distribution based on the observed numbers of deaths r and patients n at the end of the trial

Figure 1 displays the posterior distribution of treatment effect within each subset of those 4 categories according to the randomization group, suggesting possible treatment-by-subset interactions, notably with the SOFA neurological subset.

This finding prompted the following experimental design.

## Methods

### Models for binary outcomes

We considered a two-arm randomized clinical trial with a 1:1 allocation ratio. For patient *i*, let *Y*_*i*_∈{0,1} denote a binary response (where 1 denotes a non-favorable issue, such as death) and *T*_*i*_∈{0,1} denotes the treatment arm assignment, where 1 is the experimental arm and 0 the control arm. Let *θ* denote the treatment effect in the whole population that can be measured on different scales. We will use the relative risk defined by $\theta =\frac {P(Y = 1 \mid T=1)}{P(Y = 1 \mid T=0)}$ with *θ*<1 being favorable to the experimental treatment over the standard. This design would allow straightforward extension to other relative measures of treatment effect, such as odds ratios or hazard ratios.Let us consider the population partitioned into *K* subsets according to a biomarker *X* with prevalence *π*_*k*_ of subset *k*(=1,…,*K*), with $\sum \pi _{k}=1$. Let *p*_*jk*_ denote the probability of undesirable response in the *j*^*th*^ treatment arm within the *k*^*th*^ subset. In the subset *k*, let *θ*_*k*_ denote the treatment effect: $\theta _{k}=\frac {P(Y = 1 \mid T=1,X=k)}{P(Y = 1 \mid T=0,X=k)}=p_{1k}/p_{0k}.$

Following Millen [15], we considered two quantities of interest: (i) a measure of influence, i.e., treatment efficacy in the subset *k*, relying on the value of the estimation of *θ*_*k*_, and (ii) a measure of treatment-by-subset interaction. In a Bayesian setting, these two measures were considered as random variables with decision criteria expressed as posterior probabilities related to the comparison of outcomes across the arms and/or the subsets.

### Measures of influence

The influencing condition was defined as a measure of the estimated treatment effect size in each subset *k*. The posterior probability of the efficacy in subset *k* was computed as follows:

1 $$ P_{1k}=P(\theta_{k}<\lambda | Data)  $$

where *λ* defines some effect size of interest, as described by Morita [7] and more recently by Harrell to highlight the treatment effect in COVID-19 patients [17].

### Measures of interaction

Several Bayesian measures of interaction that aim to identify the sensitive subset that should be selected for the next enrollment after the interim analysis were computed. In the particular case of *K*=2 subsets, the ratio of the influence measures in both subsets, *θ*_*A*_/*θ*_*B*_, was used as the measure of interaction, as proposed by Millen [15]. The Bayesian criterion for the interaction condition was thus derived from the posterior probability of the measure and defined as follows:

2 $$  P_{2k}=P(\theta_{t}/\theta_{k}>\eta |\theta_{t} \ge \theta_{k},Data)  $$

where *η* define the minimal interaction effect and act as a threshold. We also derived a Bayesian version of the interaction statistics proposed by Gail and Simon [18]. This method facilitated the handling of greater than *K*=2 subsets and the ability to distinguish quantitative and qualitative interactions. The Gail and Simon qualitative interaction statistic was computed from the estimated log-relative risk of death in each subset *β*_*k*_ with its standard error *σ*_*k*_. It involves checking the minimum and maximum observed ratio of treatment effect over subsets:

3 $$\begin{array}{*{20}l} & Q^{-} = \sum \mathbbm{1}(\beta_{k}<0) \times (\beta_{k} / \sigma_{k})^{2} \end{array} $$

4 $$\begin{array}{*{20}l} & Q^{+} = \sum \mathbbm{1}(\beta_{k}>0) \times (\beta_{k} / \sigma_{k})^{2} \end{array} $$

The posterior probability of this statistic above the threshold *C*_1_ was derived with a qualitative interaction detected if the following criteria were met:

5 $$  P_{quali}=P\left(min\left\{Q^{-},Q^{+}\right\} > C_{1}|Data\right)  $$

The Gail and Simon quantitative interaction statistic, which is defined as the sum of differences between the estimated treatment effect in each subset *k*, *θ*_*k*_ and the global treatment effect in the trial *θ*, was also computed, and the posterior probability of this statistic was above the threshold *C*_2_ and used as a tool for decision-making as follows:

6 $$  P_{quanti}=P\left(\sum\left(\theta_{k}-\theta\right)>C_{2}|Data\right)  $$

Similarly to Gail and Simon [18], the parameters *C*_1_ and *C*_2_ were optimized through a grid search to control the false positive rate below a pre-specified level for the entire trial (Tables 1 and 2 in the supplementary materials).

### Decision rules

We propose to plan interim analyses to decide on early termination or enrichment of the trial by excluding those patients in the subsets who are not likely to satisfy the target of efficacy. Action triggers for decision-making were derived from Harrell [17], Ohwada [19] and Morita[7].

- Go with the subset *k* and stop when the interaction and the influence conditions are fulfilled in the subset *k*. As stated above, two decision criteria were assessed, based on Millen as well as Gail and Simon, respectively:
  - Interaction: according to the selected rule
    1. : (Millen) *P*_2*k*_>*τ*
    2. : (Gail and Simon) *P*_*quali*_>*ε* or/and *P*_*quanti*_>*ε*
  - Influence: *P*_1*k*_>*γ*
  where *τ*,*ε*,*γ* define decision thresholds.
- Go with the entire population regardless of the biomarker, otherwise

We applied these decision rules along the trial on the whole sample or the selected subgroups, allowing a subset with an ineffective treatment effect throughout the trial to be dropped. Note that when *K*>2, it is possible that both conditions of enrichment are met in more than one subset; thus, the trial is enriched by more than one subset simultaneously.

In both cases, the trial ended when a total of *n* patients were enrolled.

## Bayesian estimation

In each subset, we assumed that *p*_*jk*_ are Beta(*α*,*β*) distributed. Non-informative Beta(1,1) priors were first used.

Posterior distributions of *p*_*jk*_ were actualized in Beta (*α*+*y*_*jk*_,*β*+*n*_*jk*_−*y*_*jk*_) with *n*_*jk*_ the number of patients of the subpopulation *k* taking the treatment *j*. Distribution of the influence, and the interaction conditions are not straightforward.

However, given that log*θ* has been reported as normally distributed [20], we derived the measures of influence and interaction from the posterior estimator of log*θ*. Therefore, we used Markov chain Monte Carlo (MCMC) method to derive these distributions.

## Simulation study

We conducted a simulation study to examine the operating characteristics of our procedure on finite samples.

The simulation setting aimed at mimicking the motivating real trial (HIGH) regarding randomization to trial arms, treatment effects in various subsets, subset prevalence. The sample size was set at *n*=800 patients randomly allocated 1:1 to one of two randomized arms. We considered binary responses simulated across a range of scenarios corresponding to different underlying truths about the size of the treatment effect in each subset, the treatment-by-subset interaction, the prevalence of each subset and the balance of randomization in the subsets.

### Scenarios with *K*=2 subsets

We first considered *K*=2 subsets of interest, *A* and *B*, with *π*=*Pr*(*k*=*A*) denoting the prevalence of subset *A*, and *q*_*k*_=*Pr*(*T*=1|*k*), indicating the proportion of patients in the subset *k* allocated to the experimental treatment. The subsets were first considered to be well balanced in the sample due to stratification of the randomization (*q*_*A*_=*q*_*B*_=0.5), with a similar subset prevalence (*π*=0.5).

Different scenarios were simulated with varying treatment effects in each subset (Table 1). Scenario 1 refers to situations with no benefit in any subset but a pejorative prognostic value of subset *B*, scenario 2 refers to situations where there is a mild benefit in subset *A* but no effect in subset *B*, scenario 3 refers to situations where there is a marked benefit in subset *A* but no effect in subset *B*, and scenario 4 refers to situations with a large quantitative interaction (large benefit in subset *A*, but no effect in subset *B*).

**Table 1:** Description of the simulated scenarios when K = 2

Scenarios	Subset B		Subset A		Theoretical Values		
*Outcomes*	*p*_1*B*_	*p*_0*B*_	*p*_1*A*_	*p*_0*A*_	*θ*_*A*_	*θ*_*B*_/*θ*_*A*_	*RR*
Scenario 1	0.40	0.40	0.30	0.30	1.000	1.000	1.000
Scenario 2	0.40	0.40	0.30	0.40	0.750	1.330	0.875
Scenario 3	0.40	0.40	0.20	0.37	0.540	1.850	0.779
Scenario 4	0.40	0.40	0.20	0.50	0.400	2.500	0.647

Here, *p*_*jk*_ denotes the probability of death in the arm *j* in the subset *k*, and *θ*_*k*_ denotes the relative risk of death in the experimental versus the control arm in subset *k*. *RR* refers to the overall treatment effect.

### Scenarios with *K*=3 subsets

We then considered *K*= 3 subsets of interest, *A*, *B* and *C*, with *π*_*k*_=*Pr*(*k*=*k*) denoting the prevalence of subset *k*, and *q*_*k*_=*Pr*(*T*=1|*k*) indicating the proportion of patients in the subset *k* allocated to the experimental treatment. The subsets were first considered as well balanced in the sample due to stratification of the randomization (*q*_*A*_=*q*_*B*_=*q*_*C*_=0.5) with similar prevalence of the subsets ($\pi _{k}= \frac {1}{3}$). Different scenarios with varying treatment effects in each subset were considered (Table 2).

**Table 2:** Description of the simulated scenarios when *K*=3 biomarker subsets (*k*=*A*,*B*,*C*)

Scenarios	Subset A		Subset B		Subset C		Theoretical Values			
*Outcomes*	*p*_1*A*_	*p*_0*A*_	*p*_1*B*_	*p*_0*B*_	*p*_1*C*_	*p*_0*C*_	*θ*_*A*_	*θ*_*B*_	*θ*_*C*_	*RR*
Scenario 1	0.40	0.40	0.40	0.40	0.40	0.40	1.000	1.000	1.000	1.000
Scenario 2	0.32	0.40	0.32	0.40	0.32	0.40	0.800	0.800	0.800	0.800
Scenario 3	0.40	0.40	0.40	0.40	0.20	0.50	1.000	1.000	0.400	0.769
Scenario 4	0.40	0.40	0.24	0.40	0.24	0.40	1.000	0.600	0.600	0.733
Scenario 5	0.40	0.40	0.24	0.40	0.20	0.50	1.000	0.600	0.400	0.646
Scenario 6	0.40	0.40	0.24	0.40	0.50	0.40	1.000	0.600	1.250	0.925
Scenario 7	0.40	0.40	0.20	0.50	0.50	0.40	1.000	0.400	1.250	0.846

Here, *p*_*jk*_ denotes the probability of death in the arm *j* in the subset *k*, and *θ*_*k*_ denotes the relative risk of death in the experimental versus the control arm in subset *k*. *RR* refers to the overall treatment effect.

**Threshold parameters-** The minimal effect size, *λ*, was set at 0.9. For each setting (either *K*=2 or *K*=3), values of threshold parameters (*γ*,*η* and *τ*) were optimized through a grid search to maximize the power under a pre-specified value (Scenarios 2 and 4) while controlling the false positive rate under the null (Scenario 1). The false positive rate was computed as the proportion of enrichment in the subsets where there were no treatment-by-subset interaction and thus no enrichment to be made. For instance, in Scenario 1 with either *K*=2 or 3, given the similar treatment effect across all subsets, the false positive rate was defined as the proportion of observed enrichment in either subset. Details are reported in Tables and Figures in the Additional files 1.

**Sensitivity analyses-** Once the thresholds were defined, *N*=10,000 simulated trials were run for each scenario.

We then assessed the influence of the prevalence of each subset in the sample (Table 3).

**Table 3:** Description of prevalence of each subset

Number of subsets	Subset A	Subset B	Subset C
2	0.2	0.8	
	0.4	0.6	
	0.6	0.4	
	0.8	0.2	
3	1/3	1/3	1/3
	1/6	1/3	2/4
	11/18	1/3	1/18
	1/3	2/4	1/6
	1/3	1/18	11/18

Finally, we considered situations where the randomization was imbalanced in the case of K=2 by either favoring the subset where some benefit may exist or not. Thus, varying values of *q*_*A*_ that denote the proportion of participants in arm 1 in that subset, *q*_*A*_∈{0.1,0.3,0.6,0.9}, were used, whereas the value of *q*_*B*_ was computed to ensure an overall balance of the two randomized groups in the whole sample.

### Data analyses

Once the thresholds were defined simulated trials were run, for each scenario, with 3 interim analyses and one terminal analysis performed every after *n*/4=200 patients.

On each simulated dataset, we assumed that enrollment of patients was uniform over the study period in each subset. At each interim analysis, we applied the sets of rules described above. We first estimated the posterior mean of *θ*_*k*_ derived numerically from Markov chain Monte Carlo (MCMC) methods, and computed the probabilities of the previously mentioned influence and interaction measures (Eqs. 2, (6) and (5)). For the MCMC sampling procedure, we implemented three chains, with an initial burn-in of 20,000 samples followed by an additional 30,000 samples that were retained for computing *θ*_*k*_ for each chain. The first few simulation’s trace and auto-correlation plots confirmed that the chain converged, with minimal autocorrelation.

From *N*=10,000 independent replications of each trial, we calculated the proportions, over the *N* replications, of each decision (continuing the enrollment in the whole population, enriching one subset while stopping other(s)), as well as the mean number of enrolled patients in each subset. Mean influence and interaction measures, and mean biases in estimates of *θ*_*k*_, were computed over the *N* replications, with 95% credibility intervals defined by the 2.5 and 97.5 quantiles of their posterior distributions.

Regarding sensitivity analyses, we finally assessed five patterns of subset proportions *π*={*π*_1_,*π*_2_,*π*_3_} as shown in Table 3, to evaluate the sensitivity of simulation results to the subgroup prevalence. All analyses were performed using R version 4.0.1 [21] and the package “R2jags” [22].

## Results

### Detection of subset-by-treatment interaction

**Fig. 2:**
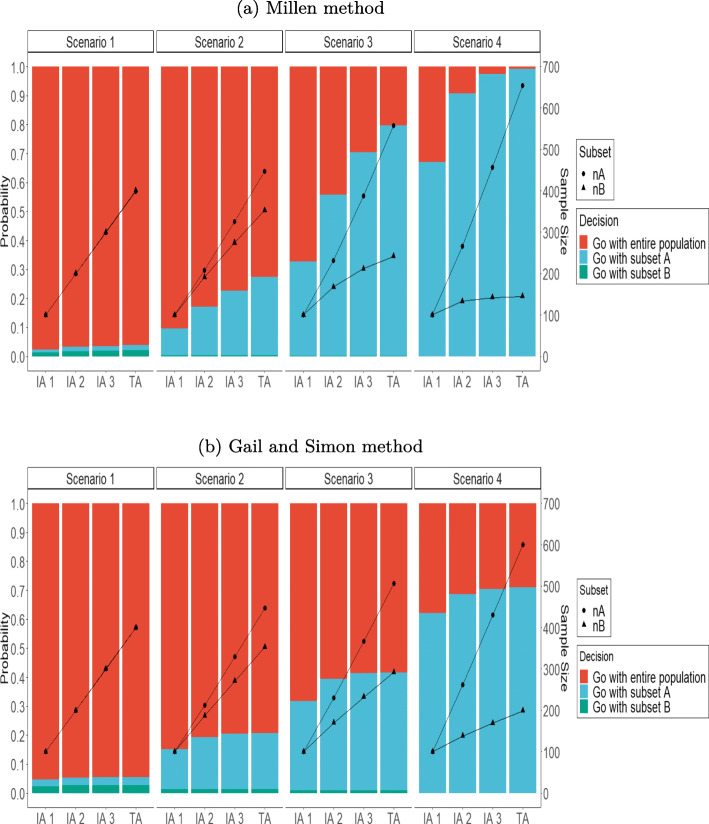
Proportions decisions and sample size along the interim and terminal analyses when K=2. *n*=800,*π*=0.5 and *q*_*B*_=*q*_*A*_=0.5. IA= Interim analysis, TA= Terminal analysis

Results of the different simulations are summarized in the Tables 4 and 5 that report the probabilities of selecting each decision over the trial according to the set of decision rules. Moreover the evolution of those decisions along the trial are shown in Fig. 2. As expected, the false positive rates in scenario 1 where no treatment effect and no treatment-by-subset interaction existed, were decreased by the sample size at the time of interim analysis (Additional file 2, Supplementary Figure 1).

**Table 4:** Comparisons of decisions at the end of the trial according to the rules when K=2

			Subset A		Subset B		
Scenarios	Interaction method	Go with EP ^∗^	go with SP (efficacy/interaction) ^∗^	*n*_*A*_	go with SP (efficacy/interaction) ^∗^	*n*_*B*_	False positive
Scenario 1	Millen	0.9622	0.0177	399.1692	0.0201	400.8308	0.0378
	Gail and Simon	0.9454	0.0282	400.2944	0.0264	399.7056	0.0546
Scenario 2	Millen	0.7259	0.2707	447.1501	0.0034	352.8499	0.0034
	Gail and Simon	0.7928	0.1934	446.8688	0.0138	353.1312	0.0138
Scenario 3	Millen	0.2025	0.7970	558.2047	0.0000	241.7953	0.0000
	Gail and Simon	0.5834	0.4080	507.3405	0.0086	292.6595	0.0086
Scenario 4	Millen	0.0067	0.9933	655.0378	0.0000	144.9622	0.0000
	Gail and Simon	0.2894	0.7106	601.0114	0.0000	198.9886	0.0000

The total sample size is set at *n*= 800, with *π*=0.5 and *q*_*B*_=*q*_*A*_=0.5.

^*^EP: entire population;

^*^SP: subpopulation (efficacy/interaction) due to the detection of interaction with efficacy in subset k;

*n*_*A*_and *n*_*B*_are the mean sample size in each subset at the end of the study

**Table 5:** Proportions of decisions at the end of the trial in seven scenarios when K=3

			Subset A		Subset B		Subset C				
Scenarios	Pattern	Go with EP ^∗^	Go with SP (efficacy/ interaction) ^∗^	*n*_*A*_	Go with SP (efficacy/ interaction) ^∗^	*n*_*B*_	Go with SP (efficacy/ interaction) ^∗^	*n*_*C*_	Go with A and B	Go with A and C	Go with B and C
Scenario 1	1	0.94	0.02	266.55	0.02	266.43	0.02	267.02	0.00	0.00	0.00
	2	0.90	0.02	137.03	0.04	268.99	0.04	393.98	0.00	0.00	0.00
	3	0.87	0.05	474.35	0.06	272.87	0.02	52.78	0.00	0.00	0.00
	4	0.89	0.04	268.64	0.04	394.67	0.02	136.68	0.00	0.00	0.00
	5	0.86	0.06	273.72	0.02	53.72	0.05	472.56	0.00	0.00	0.00
Scenario 2	1	0.87	0.04	265.96	0.04	265.86	0.04	268.18	0.00	0.00	0.00
	2	0.71	0.02	121.46	0.10	269.10	0.16	409.45	0.00	0.00	0.01
	3	0.44	0.31	480.78	0.21	279.86	0.02	39.36	0.02	0.00	0.00
	4	0.71	0.09	268.44	0.16	408.75	0.03	122.81	0.01	0.00	0.00
	5	0.46	0.21	282.57	0.01	39.47	0.30	477.95	0.00	0.01	0.00
Scenario 3	1	0.56	0.00	182.53	0.00	182.83	0.44	434.64	0.00	0.00	0.00
	2	0.09	0.00	48.59	0.00	98.19	0.90	653.22	0.00	0.00	0.00
	3	0.82	0.03	444.14	0.03	247.72	0.13	108.13	0.00	0.00	0.00
	4	0.73	0.01	218.48	0.01	328.03	0.26	253.49	0.00	0.00	0.00
	5	0.01	0.00	78.30	0.00	13.36	0.98	708.34	0.00	0.00	0.00
Scenario 4	1	0.76	0.00	222.18	0.10	289.78	0.09	288.05	0.00	0.00	0.04
	2	0.33	0.00	75.06	0.17	276.00	0.32	448.94	0.00	0.00	0.16
	3	0.46	0.02	342.21	0.50	417.39	0.02	40.39	0.00	0.00	0.00
	4	0.57	0.01	192.46	0.34	485.06	0.04	122.48	0.00	0.00	0.03
	5	0.21	0.02	145.08	0.02	34.79	0.73	620.14	0.00	0.00	0.01
Scenario 5	1	0.68	0.00	204.21	0.02	244.22	0.21	351.56	0.00	0.00	0.09
	2	0.14	0.00	52.01	0.02	183.50	0.54	564.49	0.00	0.00	0.28
	3	0.63	0.02	379.82	0.30	364.59	0.04	55.59	0.00	0.00	0.01
	4	0.68	0.00	206.75	0.13	426.00	0.09	167.25	0.00	0.00	0.08
	5	0.06	0.00	87.94	0.00	17.59	0.91	694.47	0.00	0.00	0.02
Scenario 6	1	0.67	0.01	216.19	0.31	377.23	0.00	206.58	0.00	0.00	0.00
	2	0.59	0.02	106.91	0.39	402.04	0.00	291.05	0.00	0.00	0.00
	3	0.22	0.03	296.85	0.75	476.13	0.00	27.02	0.00	0.00	0.00
	4	0.23	0.02	156.39	0.74	572.35	0.00	71.26	0.00	0.00	0.00
	5	0.84	0.05	263.31	0.11	98.39	0.00	438.31	0.00	0.00	0.00
Scenario 7	1	0.40	0.00	157.15	0.59	490.36	0.00	152.49	0.00	0.00	0.00
	2	0.30	0.00	70.66	0.70	527.77	0.00	201.56	0.00	0.00	0.00
	3	0.06	0.01	182.16	0.93	601.83	0.00	16.00	0.00	0.00	0.00
	4	0.03	0.00	87.41	0.96	671.51	0.00	41.08	0.00	0.00	0.00
	5	0.76	0.03	241.06	0.21	147.67	0.00	411.27	0.00	0.00	0.00

The total sample size is set at *n*= 800, with *π*_*k*_=1/3 and *q*_*A*_=*q*_*B*_=*q*_*C*_=0.5.

^*^EP: entire population;

^*^SP: subpopulation (efficacy/interaction) due to the detection of interaction with efficacy in subset k;

*n*_*A*_,*n*_*B*_ and *n*_*C*_ are the mean sample size in each subset at the end of the study

When *K*=2, the Gail and Simon’s interaction measure appeared more conservative than the Millen’s interaction measure. Indeed, in cases with no treatment-by-subset interaction similar to that noted in scenario 1, the design reached a false positive rate of 5.46% with the Millen’s measure and 3.78% with Gail and Simon’s measure. In scenarios 2 to 4, in which there is an increasing treatment benefit in subset A but no treatment effect in subset B, the proportion of enrichment of that subset increased from 27% in scenario 2 to 99.3% in scenario 4 using the Millen’s measure as the benefit and the sample size increased. Using the Gail and Simon measure, the proportion of enrichment in subset A increased from 19.3% to 71%. In fact, as the different scenarios progress, the greater the probability of stopping for efficacy and interaction in subset A increases, the more early the recruitment of patients in subset B is stopped.

When *K*=3, the Gail and Simon’s measure was used for decision making and we focus here on pattern 1 (where the prevalence of each subset was the same). In the case of no treatment effect in all the subsets like in scenario 1, the design reached a false positive rate of 6%. In scenario 3, which corresponds to a quantitative interaction and a very high efficacy only in subset C, the proportion of enrichment in the latter was 44%. For scenarios 4 and 5, subsets B and C had moderate or high efficacy whereas no effect was noted in subgroup A. Thus, the proportion of enrichment in scenario 4 was identical in subsets B and C. When effect was different across subsets, the design tended to recruit mainly in the subgroup with the highest efficacy as noted in scenario 5 where the proportion of enrichment in subset C reached 21%. Note that in scenarios 4 and 5, 4% and 9% of cases exhibited simultaneous enrichment in subsets B and C, respectively. Finally, in scenarios 6 and 7, which correspond to a qualitative interaction with a subgroup with no efficacy (subset A), a deleterious subset (subset C) and an effective subset (subset B), the proportion of enrichment in the subset B was slightly higher compared to the scenarios with only a quantitative interaction. Indeed, the proportion of enrichment in scenario 7 was 59% compared to 44% in scenario 3.

#### Influence of the subset prevalence

**Fig. 3:**
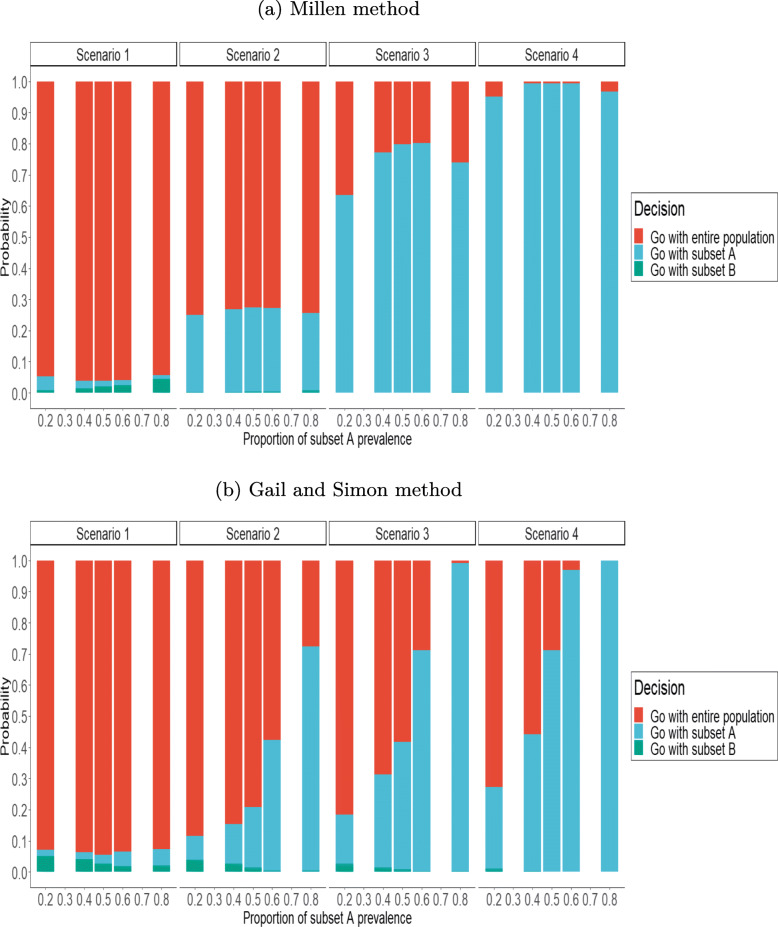
Influence of the prevalance of the subset A on the proportions decisions. *n*=800, and *q*_*B*_=*q*_*A*_=0.5 at the end of the trial

We then studied the robustness of our method for the prevalence of subset A when *K*=2. Figure 3 displays the posterior probabilities of each decision according to the set of rules for varying proportions of the prevalence of subset A in the whole sample (*π*∈{0.2,0.4,0.5,0.6,0.8}). Using Millen’s method, in scenario 1 and 2, results were poorly affected by the prevalence of subset A. In scenarios 3 and 4, the enrichment proportion in subset A was less important when the subsets were not balanced, that is, with *π*={0.2,0.8}. In fact, in scenario 3, the enrichment proportion in subset A was 63.3% with *π*=0.2, whereas it reached to 79.7% with *π*=0.5.

Concerning the Gail and Simon method, only scenario 1 was not affected by the prevalence of subset A. In scenarios 2 to 4, the enrichment proportion in subset A increased markedly with the prevalence of subset A. Indeed, with *π*=0.2, the enrichment proportion in scenario 4 was 26.3% and reached 100% with *π*=0.8.

Similarly, depending on the prevalence of subset A, the sample size in subset B decreased as the prevalence of subset A increased. Using Millen’s method with scenario 4, an average of 299 patients were recruited in subset B when *π*=0.2, but this size decreased to 66 when *π*=0.8 (Table 1 in Additional files 2).

Similarly, with *K*=3, we considered four different patterns by varying the prevalence of each subgroup in a generally balanced manner. In scenario 1, the different patterns did not affect the design decisions. However in scenarios 3 to 7, depending on the pattern used, the proportions of enrichment in the subsets varied greatly depending on whether their prevalence was high or low. Indeed, in scenario 3, the proportion of enrichment in subgroup C decreased from 44% to 13% when the prevalence of the latter decreased. Moreover, the same was true for the sample size. For example, in scenario 6, the number of patients in subgroup B was 98 with *π*_*B*_=1/18 and increased to 572 with *π*_*B*_=2/4.

#### Influence of the randomization balance

**Fig. 4:**
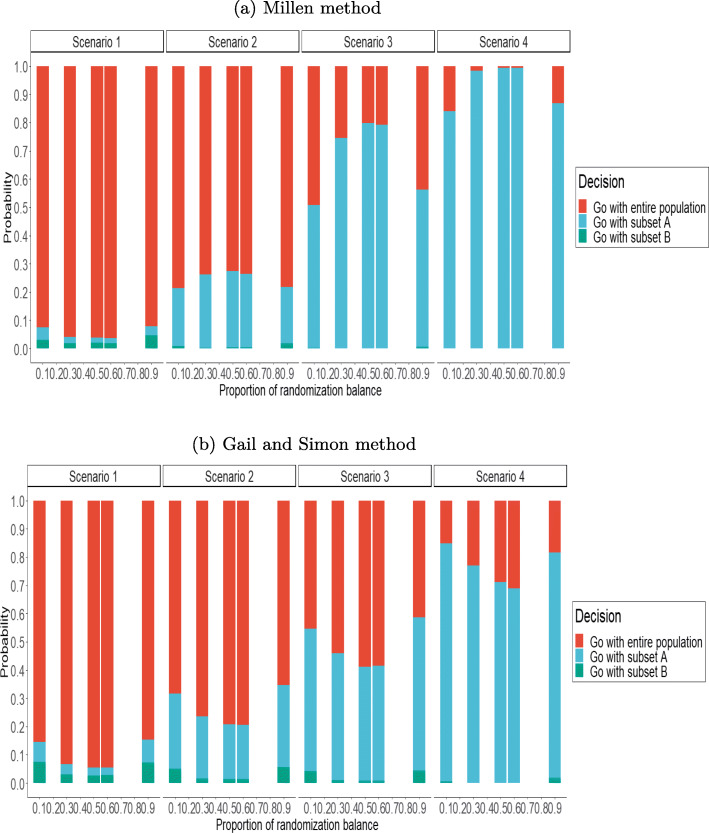
Influence of the balance of randomized group in subset A on the proportions decisions. Balance is measured by the proportion of patients in the experimental arm (*q*_*A*_) in that subset *n*=800,*π*=0.5 at the end of the trial

We similarly assessed the robustness of our findings to the balance of randomized groups in subset A, with *q*_*A*_∈{0.1,0.3,0.5,0.6,0.9} (Fig. 4).

In Scenario 1, the false positive rate was slightly greater when the randomization was imbalanced with both the methods. In fact, when *q*_*A*_={0.1,0.9}, the false positive rate was 8.5% whereas the rate was maintained under 5% in all other cases using the Millen’s method. Similary, in the case of any evidence for the treatment-by-subset interaction, results were affected by a lower detection rate in cases with large imbalances for Millen’s method. On the contrary, results were affected by and increased detection rate using the Gail and Simon method.

Indeed,the proposed design detected the treatment-by-subset interaction most often when the proportion of experimental arm in subset A was roughly balanced (between 0.3 and 0.6).

## The HIGH data set revisited: search for treatment-by-subset interactions

We retrospectively applied the proposed rules to the HIGH dataset, using the subsets of interest described above (Fig. 1). Patients were enrolled from May 19, 2016, to December 31, 2017. We considered 3 interim analyses and a terminal analysis at the following dates December 07, 2016; April 09, 2017; September 01, 2017; and December 31, 2017. These dates were retrospectively chosen according to the recruitment, to perform the interim analysis every time 194 new patients were enrolled if no prior decision of enrichment was made. In the case of detection of treatment-by-subset interaction, enrichment towards the subset with detected efficacy and interaction was performed thereafter with no further evaluation of interaction in subsequent analyses, unless there were no more available observations in the sample, while stopping enrollment from other subsets. Similarly to the Simulation study, threshold parameters of the rules were set to minimize the false positive rate of detecting interaction.

Results are summarized in Tables 6 and 7. Some evidence of a qualitative interaction with age and a quantitative interaction with the SOFA Neurological partition (Fig. 1) was noted. A treatment-by-subset interaction was highlighted in the neurological SOFA partition, as observed in Fig. 1. This was detected since the second interim analysis, where the Millen’s efficacy and interaction conditions, as well as the Gail and Simon’s criterion, were verified in the subset where the SOFA neurological score was greater than 1. Thus, following this analysis, no more inclusions were considered in subset with neurological SOFA <1, while enrichment in those with neurological SOFA ≥1 resulted in the only inclusion of new patients from this later subset whichever the rule.

**Table 6:** HIGH clinical trial: Detection of treatmen-by-subset interaction when K=2

	Interaction method	$\boldsymbol {\widehat {\theta }}$ global CI95%	$\boldsymbol {\widehat {\theta _{A}}}$ CI95%	$\boldsymbol {\widehat {\theta _{B}}}$ CI95%	Decision	*n*_*A*_	*n*_*B*_	Probability of interaction effect*
**Age Partition**			*Age*≥65	*Age*<65				
1er interim analysis	Millen		1.228[0.721-1.988]	0.913[0.548-1.412]	Go with entire population	87	107	0.56
	Gail and Simon		1.228[0.721-1.988]	0.913[0.548-1.412]	Go with entire population	87	107	0.02& 0.09
2nd interim analysis	Millen		1.367[0.931-1.957]	0.868[0.586-1.241]	Go with entire population	178	210	0.80
	Gail and Simon		1.367[0.931-1.957]	0.868[0.586-1.241]	Go with entire population	178	210	0.02& 0.08
3th interim analysis	Millen		1.184[0.879-1.568]	0.745[0.529-1.011]	Go with entire population	265	320	0.85
	Gail and Simon		1.184[0.879-1.568]	0.745[0.529-1.011]	Go with entire population	265	320	0.01& 0.00
Final analysis	Millen	0.989[0.819-1.182]	1.245[0.956-1.597]	0.780[0.580-1.017]	Go with entire population	360	416	0.9
	Gail and Simon	0.989[0.819-1.182]	1.245[0.956-1.597]	0.780[0.580-1.017]	Go with entire population	360	416	0.01& 0.00
**SOFA neurological Partition**			*SOFA* *neuro*≥1	*SOFA* *neuro*<1				
1er interim analysis	Millen		0.799[0.391-1.349]	1.136[0.750-1.655]	Go with entire population	27	167	0.66
	Gail and Simon		0.799[0.391-1.349]	1.136[0.750-1.655]	Go with entire population	27	167	0.02& 0.02
2nd interim analysis	Millen		0.633[0.341-1.026]	1.252[0.916-1.677]	Enrichment in subset A	54	334	0.94
	Gail and Simon		0.633[0.341-1.026]	1.252[0.916-1.677]	Enrichment in subset A	54	334	0.08& 0.00
3th interim analysis	Millen		0.753[0.477-1.107]	1.252[0.916-1.679]	Enrichment in subset A	77	334	–
	Gail and Simon		0.753[0.477-1.107]	1.252[0.916-1.679]	Enrichment in subset A	77	334	–
Final analysis	Millen	1.156[0.890-1.477]	0.858[0.350-1.688]	1.252[0.916-1.677]	Enrichment in subset A	102	334	–
	Gail and Simon	1.156[0.890-1.477]	0.858[0.350-1.688]	1.252[0.916-1.677]	Enrichment in subset A	102	334	–
***PaO***_***2***_***/FiO***_***2***_**Partition**			***PaO***_***2***_***/FiO***_***2***_<100	***PaO***_***2***_***/FiO***_***2***_≥100				
1er interim analysis	Millen		1.380[0.852-2.131]	0.848[0.498-1.342]	Go with entire population	80	114	0.78
	Gail and Simon		1.380[0.852-2.131]	0.848[0.498-1.342]	Go with entire population	80	114	0.07& 0.28
2nd interim analysis	Millen		1.164[0.790-1.659]	1.050[0.717-1.475]	Go with entire population	152	236	0.30
	Gail and Simon		1.164[0.790-1.659]	1.050[0.717-1.475]	Go with entire population	152	236	0.00& 0.03
3th interim analysis	Millen		0.973[0.694-1.319]	0.957[0.711-1.254]	Go with entire population	219	366	0.16
	Gail and Simon		0.973[0.694-1.319]	0.957[0.711-1.254]	Go with entire population	219	366	0.00& 0.00
Final analysis	Millen	0.998[0.824-1.196]	0.983[0.731-1.292]	1.017[0.809-1.263]	Go with entire population	278	498	0.09
	Gail and Simon	0.998[0.824-1.196]	0.983[0.731-1.292]	1.017[0.809-1.263]	Go with entire population	278	498	0.00& 0.00

The reported intervals are 95% credibility intervals, defined as [quantile(2.5*%*), quantile(97.5*%*)] of the posterior distribution.

* In case of Millen’s criterion, this refers to the posterior probability that *P*_2*K*_ (equation (2)). In case of Gail & Simon’s criterion, it refers to the posterior probabilities *P*_*quali*_ and *P*_*quanti*_, respectively, as described in equations (5) & (6).

**Table 7:** HIGH clinical trial: Detection of treatmen-by-subset interaction when K=3

	$\boldsymbol {\widehat {\theta }}$**global CI95%**	$\boldsymbol {\widehat {\theta _{A}}}$**CI95%**	$\boldsymbol {\widehat {\theta _{B}}}$**CI95%**	$\boldsymbol {\widehat {\theta _{C}}}$**CI95%**	Decision	*n*_*A*_	*n*_*B*_	*n*_*C*_	Proportion interaction effect*
**Age Partition**	*Age*≤58	58<*Age*≤68	*Age*>68						
1er interim analysis		1.062[0.484-1.972]	0.804[0.444-1.280]	1.558[0.806-2.969]	Go with entire population	61	74	59	0.21& 0.46
2nd interim analysis		0.913[0.518-1.493]	0.979[0.612-1.450]	1.437[0.920-2.217]	Go with entire population	127	135	126	0.10& 0.12
3th interim analysis		0.755[0.475-1.108]	0.975[0.670-1.381]	1.086[0.782-1.485]	Go with entire population	197	202	186	0.01& 0.00
Final analysis	0.993[0.823-1.18]	0.850[0.570-1.209]	0.986[0.719-1.334]	1.141[0.838-1.499]	Go with entire population	255	268	253	0.00& 0.00

The reported intervals are 95% credibility intervals, defined as [quantile(2.5*%*), quantile(97.5*%*)] of the posterior distribution.

^*^In case of Gail & Simon, it refers to the posterior probabilities *P*_*quali*_ and *P*_*quanti*_, respectively, as described in equation (5) & (6).

Concerning the age partition (with 2 subsets), none of our decision rules were fulfilled. In fact, at the 3rd interim analysis, the posterior probabilities of the influence and interaction conditions were 0.86 and 0.89, respectively, for the subset of patients aged 65 years or less with Millen method.The same results have been obtained by Gail and Simon’s criterion. These values are less than but close to the threshold parameters established at 0.90 for efficacy and interaction. For the age partition with 3 subsets, the condition of interaction is only verified for the two first interim analyses while the condition of efficacy was never satisfied throughout the trial.

## Discussion

An adaptive design is a clinical trial design that allows adaptations or modifications to some aspects of the trial after its initiation without undermining the validity of the trial [23]. Many adaptations have been proposed, including biomarkers-based trials that use information obtained from classifier biomarkers (that is, markers defined at baseline that do not change over the course of the study). Thus, we aimed at providing some Bayesian enrichment adaptive designs for randomized clinical trials, focusing on restricting the inclusion to the subset of patients who are likely to benefit the most from the treatment during the trial accrual, as previously reported [6, 8]. This method should offer the potential to reduce the risks and the costs of drug development and bring much needed new medicines to those patients with greater efficiency. In addition, the patients enrolled in the trial also benefit. Such adaptive enrichment designs may vastly increase power, especially when only a small subset of patients drive treatment response [8]. Nevertheless, this obviously impacts the overall estimate of the treatment effect, which is no longer relevant, and this is the reason why it was even not reported at all.

We combined previously published Bayesian rules based on efficacy [7] and interaction [15] measures to that end. Furthermore, we proposed a Bayesian version of the Gail and Simon interaction statistics [18] as a measure of interaction for our decision rules, allowing the extension of this design to more than two subsets. Our design differs from previously published Wang’s patient enrichment design and Liu’s threshold enrichment design as the treatment effect is estimated for each subset simultaneously from the first stage. Moreover, it focuses solely on enrichment in a perspective of personalized medicine [24, 25]. Likewise, our design appears close to the adaptive enrichment design proposed by Xia *et al* [26] who also proposed a signature enrichment design with adaptive randomization; nevertheless, their use of an enrichment strategy together with a Bayesian adaptive randomization scheme, adds complexities compared to our design. Such a complexity could be also pointed out in the recent proposal from Ballarini et al. [27] who proposed a Bayesian optimization for a two-stage design, using some utility function taking into account the prevalence of the subsets. Our design appear to be more easily understood by practitioners.

The Bayesian framework, allows the incorporation of previous information, if any, into the analyses and using probabilistic statements regarding efficacy as decision criteria, as recently exemplified in emergency randomized trials [28] and the COVID-19 pandemic [29–31]. The Bayesian paradigm allows the incorporation of the investigator intuitions through prior distribution.

Enrichment designs typically consist of two or three stages, where the first stage serves as a screening process for selecting a certain subpopulation, and the succeeding stages serve to distinguish the treatment effect from the placebo effect within the selected (enriched) subpopulation [32,33]. We placed ourselves in a more integrative setting, where the two stages are indeed considered sequentially in the same trial. We indeed provided a potential reallocation of scheduled patients to the single subset more likely to benefit from the intervention from a two-parallel arm RCT.

In the case of *K*=2, Millen’s approach was more sensitive than the Gail & Simon’s statistic to the difference of treatment effect between the subsets, which means that when the treatment effect was increasingly different across the subsets, and thus the interaction increased, Millen’s method tended to enrich more frequently and quickly the sample from the subset of interest. Note also that Millen’s rules were more sensitive to the sample size at each interim analysis as observed in Fig. 2. Indeed, the proportion of enrichment in the subset of interest at the first interim analysis with two hundred patients was similar with both methods although at further interim analyses where the number of patients increased, the enrichment proportion was much important with the Millen’s approach. However, the Gail and Simon’s interaction measure was also satisfying, and it’s the only option when there are more than two subsets of interest. When the randomization was stratified on the subsets, resulting in balanced treatment arms among each subset (as illustrated by *q*_*A*_=*q*_*B*_=0.5), the results showed the best performances when selecting the right subset. Millen’s measure of interaction appeared more robust than the Gail and Simon’s method due to imbalances of randomization within the subset of interest and the prevalence of the subsets. As observed in sequential trials [34], we think that these rules should not be applied too early, unless the sample size was sufficiently large for decision-making.

### Limitations

Our study has some limitations. We only considered categorization of the whole population into two non-overlapping subsets. However, given that the biomarkers of interest are often not clear binary variables, this raises the issues of selecting the cutoff, and how to combine several biomarkers to define such a partition. Besides, we assumed a uniform enrollment of patient in our simulation study, an assumption which is likely violated in many actual clinical trial settings. If the interim analyses take place at fixed time periods, the violation of this assumption may impact the operating characteristics of the design. Indeed, if the number of patients enrolled at the first analyses is lower or higher than expected, it can cause the trial to be under-powered or over-powered, respectively [35]. On the contrary, if the interim analyses occur when prespecified fixed numbers of patients have been enrolled, the findings will not be impacted by the recruitment rate, as the information time of each analysis is the one expected [36]. Thus, we recommended to schedule the interim analyses when fixed numbers of patients have been reached to avoid any impact of the recruitment process. We only used a binary outcome though it could be extended to a survival outcome using hazard ratios. A Bayesian group sequential enrichment design has been recently proposed [37]. It uses a joint probability model for both the response and the survival outcomes. However, the method requires many design parameters, requiring sample sizes of several hundred patients. Moreover, it requires computations that not straightforward compared to our proposal that appears more easily interpreted by clinicians. In our illustration based on the HIGH trial data, the choice of subsets could appear somewhat poorly substantiated by clinical hypotheses, and other subsets such those based on the cause of the ARF or on the existence of an underlying sepsis, may have had been chosen. The main point is that in any case, this choice should be prespecified in the protocol to avoid any “fishing expedition”. At last, not at least, although results of the simulation study argued that one may use the enrichment design with good properties regarding the control of false positive detection, it is likely that its use in practice could be delayed, as exemplified in other settings by Robert Altman more than 25 years ago [38]. Thus, we also schedule to use such methods in real randomized clinical trials to exemplify their interests in practice.

### Future directions

It could be useful to extend the design to other situations, such as studies with a higher number of non-overlapping subpopulations or those with overlapping and even nested subpopulations.

## Conclusion

In the next few years, the need for personalized medicine is likely to continue to increase with a growing demand for adaptive enrichment designs that handle the uncertainty in treatment efficacy by focusing on the target population. Given this need for designs that allow rapid answers to therapeutic questions, such enrichment designs may appear of interest to avoid the waste of research, notably in the settings where the population is known to be heterogeneous with potential different responses to the treatment. Our proposed strata-based design offers a promising balance for trial efficiency, and ease of interpretation.

## Supplementary Information

**Additional file 1** Simulated operating characteristics for the threshold parameters for Bayesian adaptive design

**Additional file 2** Simulated sensitivity analysis for Bayesian adaptive design when K=2

## Data Availability

The data and computer code used to analyse the motivating example, the computer code used to create and analyse the simulated data sets, and the computer code used to plot the figures and tables in the manuscript (including Additional files) are available in the github repository, https://github.com/vvinnat/Enrichment-Bayesian-design-with-multiple-classifier-biomarkers.
