# Supplementary material for: Enrichment Bayesian design for randomized clinical trials using categorical biomarkers and a binary outcome
Source: BMC Med Res Methodol. 2022 Feb 27;22:54. doi: 10.1186/s12874-022-01513-z (PMC8882316; doi:10.1186/s12874-022-01513-z)
Supplement: Supplementary file 1 — Additional file 1 Simulated operating characteristics for the threshold parameters for Bayesian adaptive design [file 12874_2022_1513_MOESM1_ESM.pdf]

Additional file 1: Simulated operating characteristics for the threshold parameters for Bayesian adaptive design

Table 1: Critical values (C1, C2) for the Bayesian version of Gail and Simon Test

**Number of subgroups**		**False positive rate**			
		**0.20**	**0.15**	**0.10**	**0.05**
**2**	Quantitative	0.045	0.070	0.105	0.220
	Qualitative	0.060	0.110	0.210	0.655
**3**	Quantitative	0.155	0.230	0.360	0.710
	Qualitative	0.325	0.505	0.980	1.955

Table 2: Threshold values defined through grid search

Interaction measure	K	γ	η	τ	ε
Millen	2	0.90	1.25	0.90	
Gail & Simon	2	0.90			0.05
	3	0.90			0.05

Figure 1: Critical values (C1, C2) for the Bayesian version of the Gail and Simon Test when K=2

(a) Quantitative

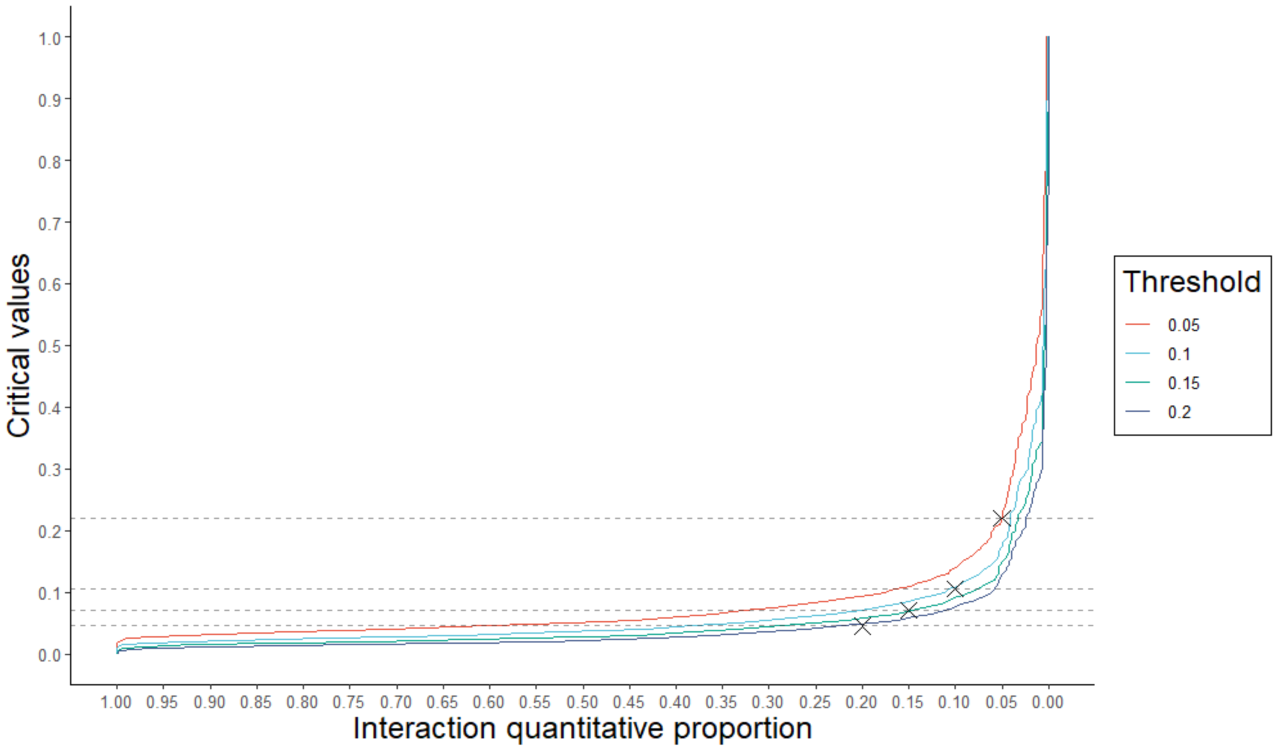

(b) Qualitative

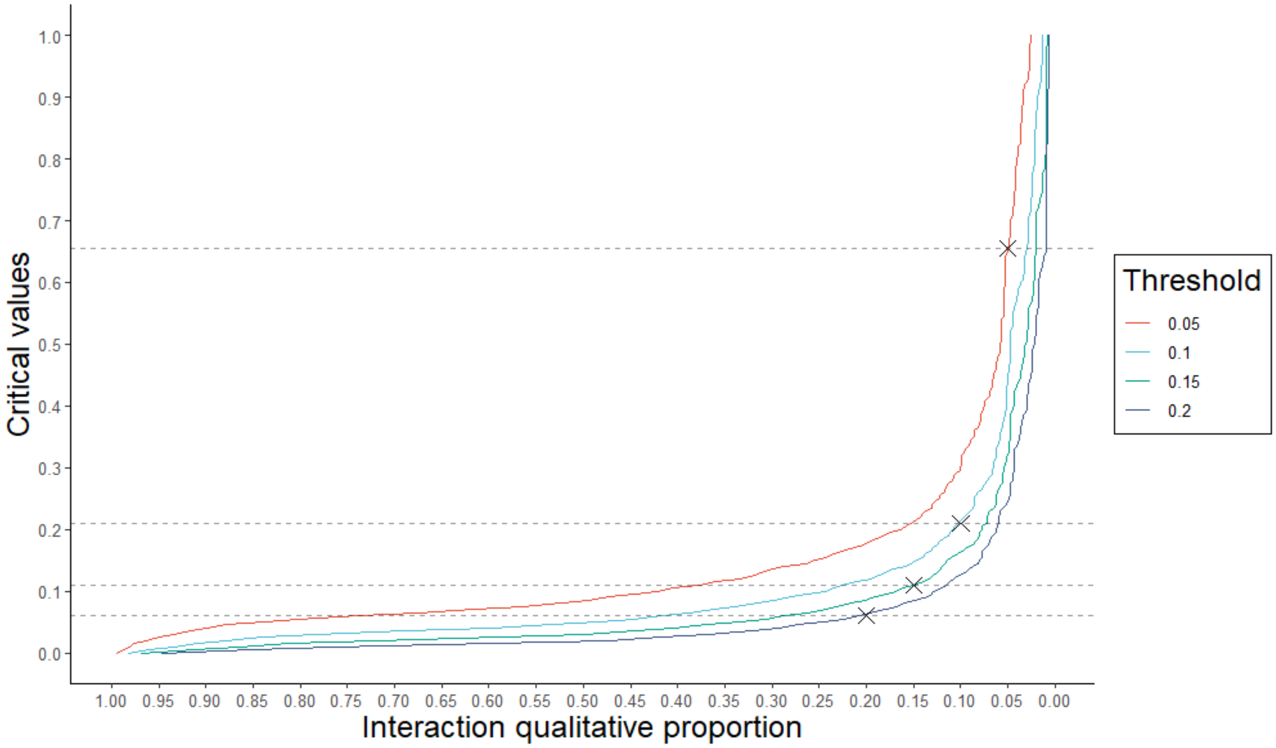

The x-axis corresponds to the probabilities defined in equations 5 and 6 in the main manuscript.

Figure 2: Critical values (C1, C2) for the Bayesian version of the Gail and Simon Test when K=3

(a) Quantitative

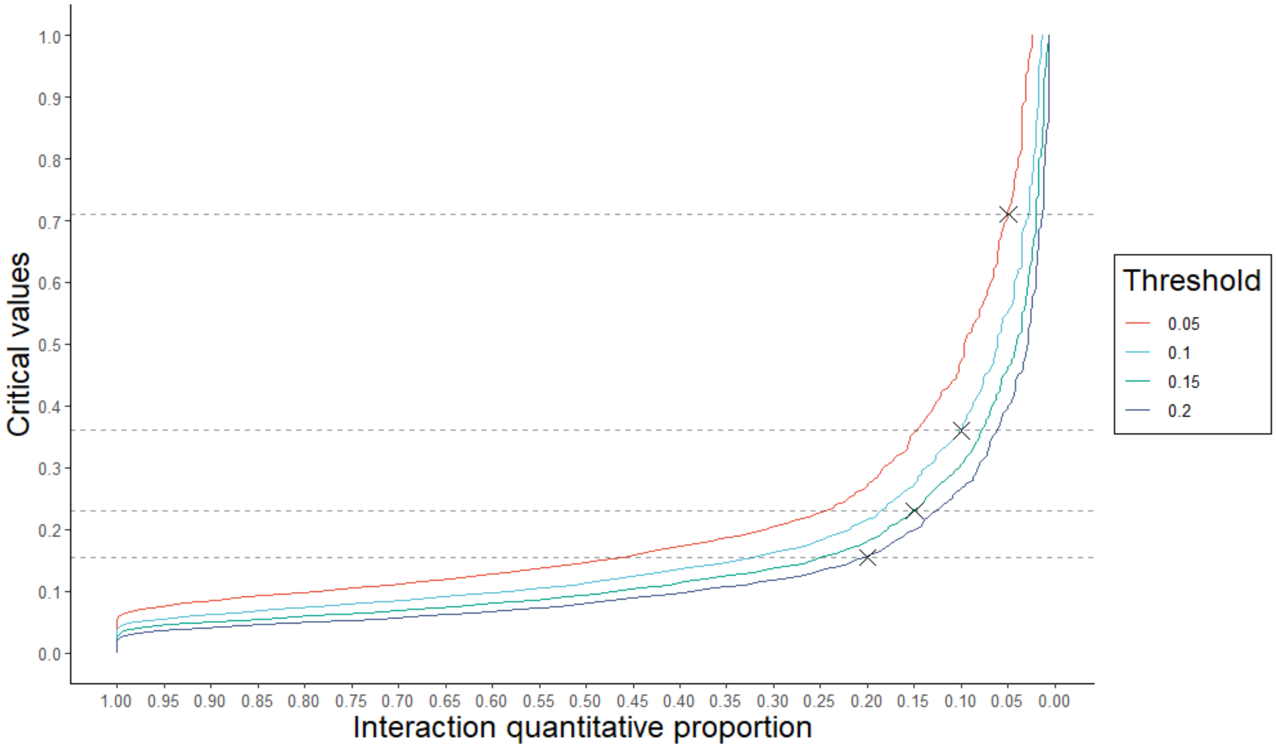

(b) Qualitative

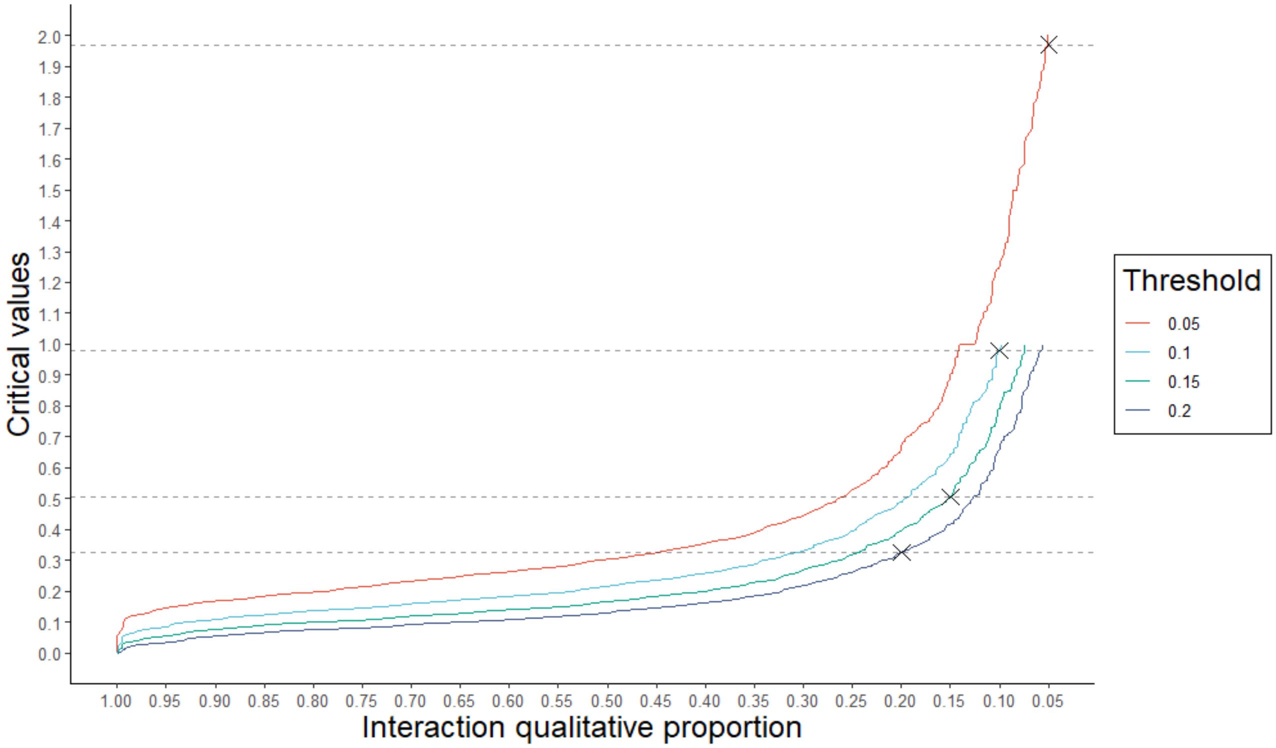

The x-axis corresponds to the probabilities defined in equations 5 and 6 in the main manuscript.

Figure 3: Proportion of false positive rates in the scenario 1 when K=2.

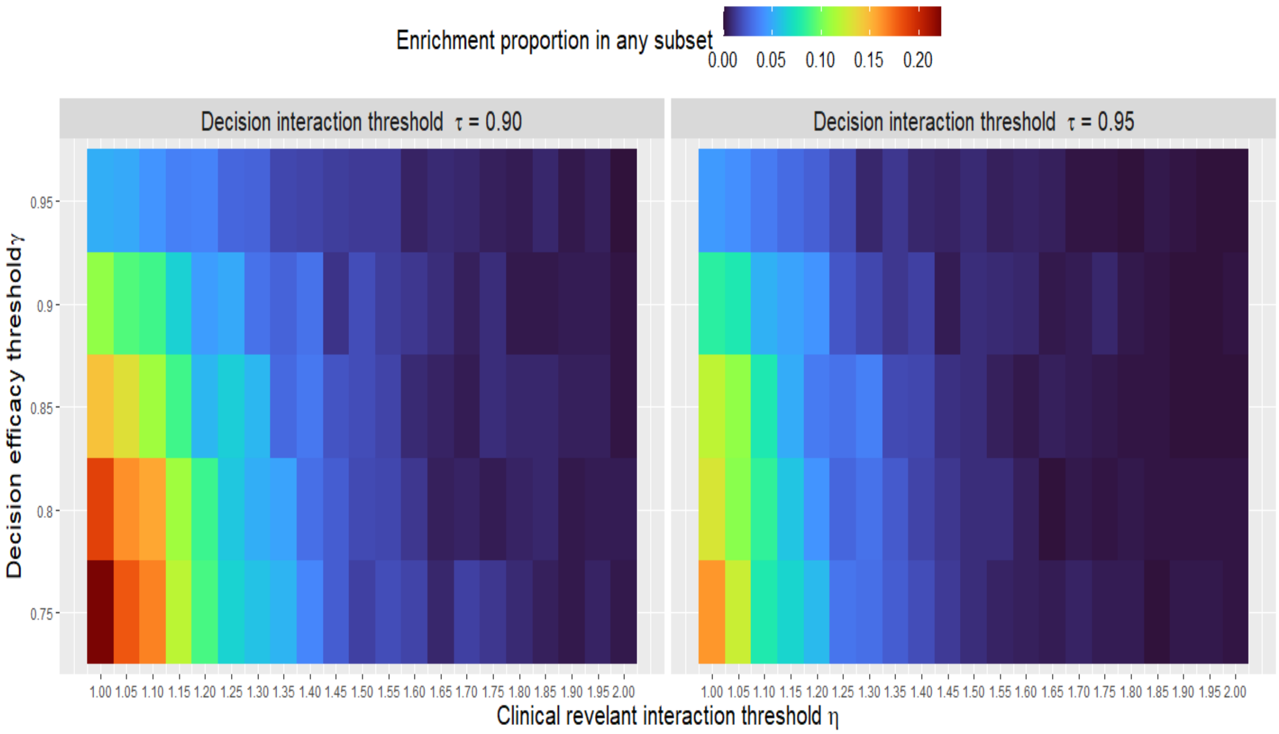

with $\lambda = 0.9$, according to different values of γ, η, and τ.

Figure 4: Proportion of true positive rates in the scenario 2 when K=2.

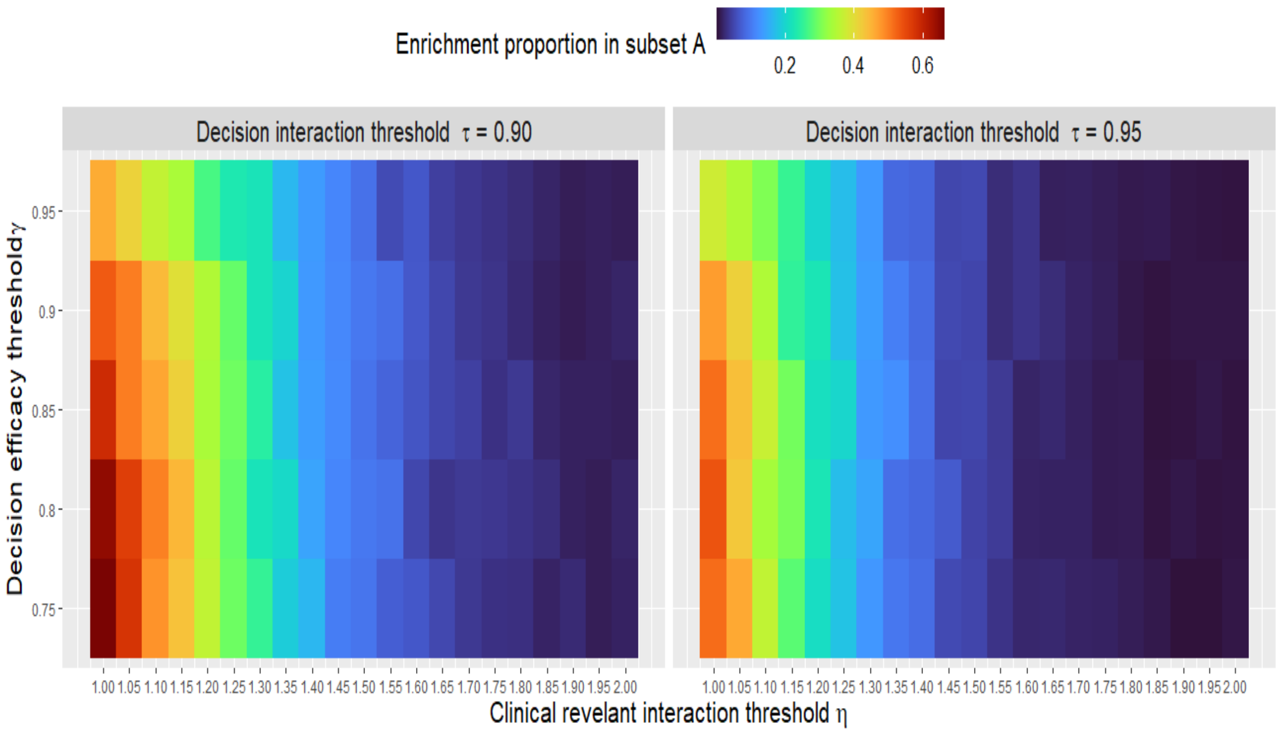

with $\lambda = 0.9$, according to different values of γ, η, and τ.

Figure 5: Proportion of true positive rates in the scenario 4 when K=2.

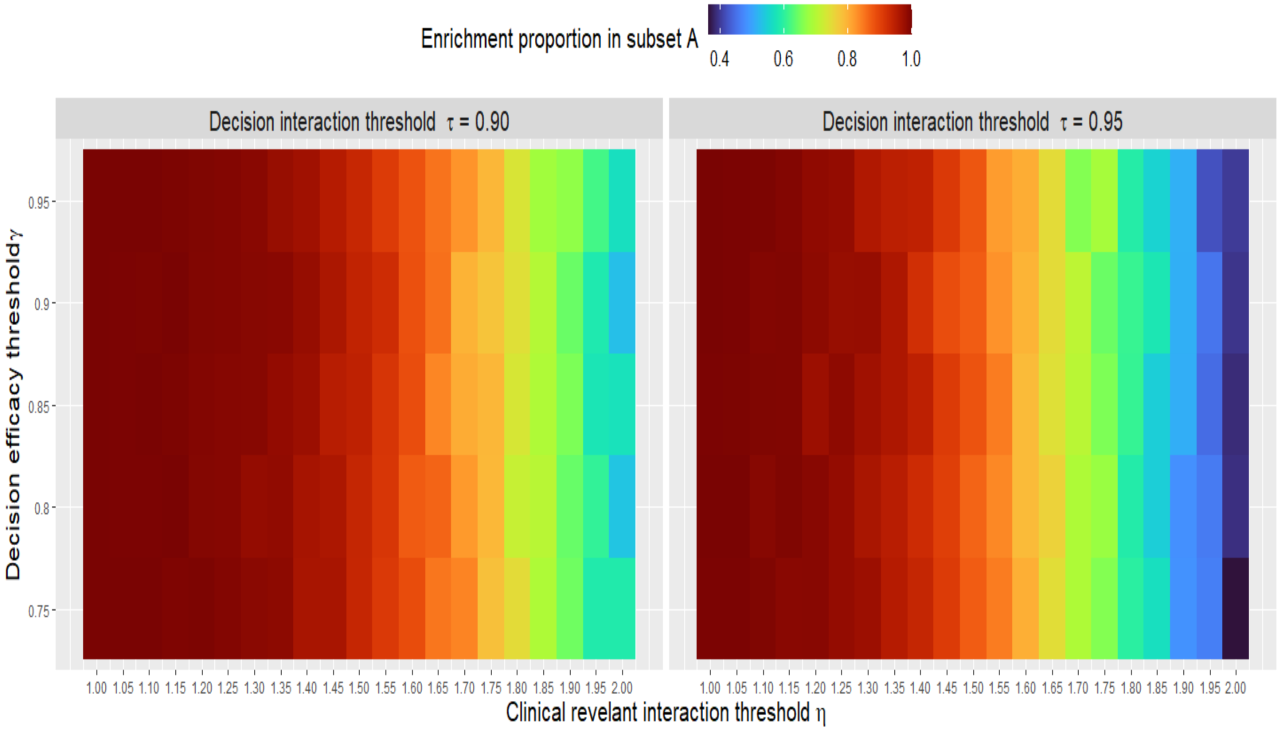

with $\lambda = 0.9$, according to different values of γ, η, and τ.
