# Supplementary material for: Enrichment Bayesian design for randomized clinical trials using categorical biomarkers and a binary outcome
Source: BMC Med Res Methodol. 2022 Feb 27;22:54. doi: 10.1186/s12874-022-01513-z (PMC8882316; doi:10.1186/s12874-022-01513-z)
Supplement: Supplementary file 2 — Additional file 2 Simulated sensitivity analysis for Bayesian adaptive design when K=2 [file 12874_2022_1513_MOESM2_ESM.pdf]

Additional file 2: Simulated sensitivity analysis for Bayesian adaptive design when K=2

Table 1: Influence of the prevalence of the subset on the proportions decisions when K=2.

			Subset A			Subset B		
**Scenarios**	**Interaction method**	π	**Go with EP***	**go with SP (efficacy/interaction)***	$\mathbf{n_A}$	**go with SP (efficacy/interaction)***	$\mathbf{n_B}$	**biais of θ_A(sd)**
Scenario 1	Millen	0.2	0.9466	0.0447	176.3886	0.0087	623.6114	0.0537(0.2087)
	Gail and Simon		0.9301	0.0200	164.3623	0.0499	635.6377	0.0447(0.2147)
	Millen	0.4	0.9622	0.0232	323.7105	0.0146	476.2895	0.0261(0.1437)
	Gail and Simon		0.9379	0.0206	318.6311	0.0415	481.3689	0.0239(0.1462)
	Millen	0.5	0.9622	0.0177	399.1692	0.0201	400.8308	0.0217(0.1284)
	Gail and Simon		0.9454	0.0282	400.2944	0.0264	399.7056	0.0200(0.1292)
	Millen	0.6	0.9594	0.0156	475.8357	0.0250	324.1643	0.0191(0.1173)
	Gail and Simon		0.9358	0.0448	482.5765	0.0194	317.4235	0.0179(0.1165)
	Millen	0.8	0.9438	0.0116	624.8981	0.0446	175.1019	0.0148(0.1022)
	Gail and Simon		0.9276	0.0520	635.9898	0.0204	164.0102	0.0133(0.1007)
Scenario 2	Millen	0.2	0.7497	0.2483	234.2336	0.0020	565.7664	0.0577(0.1654)
	Gail and Simon		0.8855	0.0767	190.0942	0.0378	609.9058	0.0465(0.1770)
	Millen	0.4	0.7325	0.2654	378.9182	0.0021	421.0818	0.0280(0.1149)
	Gail and Simon		0.8459	0.1286	355.5484	0.0255	444.4516	0.0262(0.1199)
	Millen	0.5	0.7259	0.2707	447.1501	0.0034	352.8499	0.0211(0.1034)
	Gail and Simon		0.7928	0.1934	446.8688	0.0138	353.1312	0.0215(0.1049)
	Millen	0.6	0.7272	0.2692	517.3640	0.0036	282.6360	0.0171(0.0948)
	Gail and Simon		0.5767	0.4183	554.8692	0.0050	245.1308	0.0190(0.0925)
	Millen	0.8	0.7433	0.2475	653.6661	0.0092	146.3339	0.0115(0.0833)
	Gail and Simon		0.2755	0.7209	696.3877	0.0036	103.6123	0.0127(0.0807)
Scenario 3	Millen	0.2	0.3661	0.6334	348.0001	0.0000	451.9999	0.0573(0.1261)
	Gail and Simon		0.8160	0.1580	226.6031	0.0260	573.3969	0.0494(0.1518)
	Millen	0.4	0.2290	0.7706	501.2211	0.0000	298.7789	0.0273(0.0894)
	Gail and Simon		0.6877	0.2977	412.7852	0.0146	387.2148	0.0257(0.0999)
	Millen	0.5	0.2025	0.7970	558.2047	0.0000	241.7953	0.0203(0.0820)
	Gail and Simon		0.5834	0.4080	507.3405	0.0086	292.6595	0.0197(0.0872)
	Millen	0.6	0.1973	0.8020	608.5261	0.0000	191.4739	0.0170(0.0772)
	Gail and Simon		0.2878	0.7095	627.9041	0.0027	172.0959	0.0160(0.0763)
	Millen	0.8	0.2599	0.7386	697.8912	0.0015	102.1088	0.0111(0.0706)
	Gail and Simon		0.0079	0.9908	745.2749	0.0017	54.7251	0.0104(0.0681)
Scenario 4	Millen	0.2	0.0487	0.9512	500.1862	0.0000	299.8138	0.0234(0.0644)
	Gail and Simon		0.7275	0.2631	278.2111	0.0094	521.7889	0.0282(0.0936)
	Millen	0.4	0.0069	0.9931	620.4717	0.0000	179.5283	0.0096(0.0523)
	Gail and Simon		0.5586	0.4391	469.7840	0.0023	330.2160	0.0123(0.0629)
	Millen	0.5	0.0067	0.9933	655.0378	0.0000	144.9622	0.0081(0.0504)
	Gail and Simon		0.2894	0.7106	601.0114	0.0000	198.9886	0.0093(0.0537)
	Millen	0.6	0.0066	0.9934	684.2338	0.0000	115.7662	0.0075(0.0491)
	Gail and Simon		0.0297	0.9703	702.2053	0.0000	97.7947	0.0064(0.0483)
	Millen	0.8	0.0320	0.9678	733.6633	0.0000	66.3367	0.0071(0.0473)
	Gail and Simon		0.0000	1.0000	759.0251	0.0000	40.9749	0.0054(0.0462)

The total sample size is set at n= 800, with $\pi \in \{0.2, 0.4, 0.6, 0.8\}$ and $q_B = q_A = 0.5$.
*EP: entire population;
*SP: subpopulation (efficacy/interaction) due to the detection of an interaction with efficacy in subset k;
n_A and n_B are the mean sample size in each subset at the end of the study

Table 2: Influence of the balance of randomized group on the proportions decisions when K=2

			Subset A			Subset B		
**Scenarios**	**Interaction method**	**q1**	**Go with EP***	**go with SP (efficacy/interaction)***	$\mathbf{n_A}$	**go with SP (efficacy/interaction)***	$\mathbf{n_B}$	**biais of θ_A(sd)**
Scenario 1	Millen	0.1	0.9248	0.0451	403.4763	0.0301	396.5237	0.0305(0.2024)
	Gail and Simon		0.8548	0.0710	397.9140	0.0742	402.0860	0.0312(0.2084)
	Millen	0.3	0.9585	0.0227	401.1200	0.0188	398.8800	0.0200(0.1369)
	Gail and Simon		0.9338	0.0353	400.7344	0.0309	399.2656	0.0191(0.1385)
	Millen	0.5	0.9622	0.0177	399.1692	0.0201	400.8308	0.0217(0.1284)
	Gail and Simon		0.9454	0.0282	400.2944	0.0264	399.7056	0.0200(0.1292)
	Millen	0.6	0.9633	0.0181	399.6886	0.0186	400.3114	0.0245(0.1326)
	Gail and Simon		0.9452	0.0273	399.8326	0.0275	400.1674	0.0235(0.1337)
	Millen	0.9	0.9214	0.0325	396.3688	0.0461	403.6312	0.1181(0.5858)
	Gail and Simon		0.8461	0.0820	403.9753	0.0719	396.0247	0.1254(0.5467)
Scenario 2	Millen	0.1	0.7872	0.2046	434.4907	0.0082	365.5093	0.0418(0.1813)
	Gail and Simon		0.6832	0.2659	439.1489	0.0509	360.8511	0.0468(0.1860)
	Millen	0.3	0.7374	0.2594	446.1276	0.0032	353.8724	0.0234(0.1158)
	Gail and Simon		0.7634	0.2202	449.7169	0.0164	350.2831	0.0236(0.1171)
	Millen	0.5	0.7259	0.2707	447.1501	0.0034	352.8499	0.0211(0.1034)
	Gail and Simon		0.7928	0.1934	446.8688	0.0138	353.1312	0.0215(0.1049)
	Millen	0.6	0.7356	0.2606	446.1862	0.0038	353.8138	0.0238(0.1048)
	Gail and Simon		0.7943	0.1906	445.9297	0.0151	354.0703	0.0234(0.1062)
	Millen	0.9	0.7832	0.1982	431.1679	0.0186	368.8321	0.0934(0.4841)
	Gail and Simon		0.6534	0.2898	454.9315	0.0568	345.0685	0.1062(0.6503)
Scenario 3	Millen	0.1	0.4925	0.5053	485.4763	0.0022	314.5237	0.0556(0.1647)
	Gail and Simon		0.4541	0.5034	491.0982	0.0425	308.9018	0.0594(0.1697)
	Millen	0.3	0.2553	0.7444	541.9958	0.0000	258.0042	0.0256(0.0968)
	Gail and Simon		0.5410	0.4478	510.9485	0.0112	289.0515	0.0239(0.1011)
	Millen	0.5	0.2025	0.7970	558.2047	0.0000	241.7953	0.0203(0.0820)
	Gail and Simon		0.5886	0.4028	506.1640	0.0086	293.8360	0.0197(0.0873)
	Millen	0.6	0.2072	0.7922	557.8027	0.0000	242.1973	0.0214(0.0813)
	Gail and Simon		0.5857	0.4050	506.3845	0.0093	293.6155	0.0210(0.0867)
	Millen	0.9	0.4386	0.5559	499.4974	0.0055	300.5026	0.0671(0.2642)
	Gail and Simon		0.4142	0.5421	520.9427	0.0437	279.0573	0.0877(0.4726)
Scenario 4	Millen	0.1	0.1603	0.8395	567.9415	0.0000	232.0585	0.0348(0.1107)
	Gail and Simon		0.1528	0.8407	599.8419	0.0065	200.1581	0.0313(0.1079)
	Millen	0.3	0.0177	0.9823	640.3151	0.0000	159.6849	0.0129(0.0620)
	Gail and Simon		0.2311	0.7687	613.2497	0.0000	186.7503	0.0113(0.0639)
	Millen	0.5	0.0067	0.9933	655.0378	0.0000	144.9622	0.0081(0.0504)
	Gail and Simon		0.2894	0.7106	601.0114	0.0000	198.9886	0.0090(0.0537)
	Millen	0.6	0.0058	0.9942	653.4604	0.0000	146.5396	0.0082(0.0486)
	Gail and Simon		0.3110	0.6886	595.2088	0.0000	204.7912	0.0099(0.0522)
	Millen	0.9	0.1318	0.8670	579.0985	0.0012	220.9015	0.0279(0.0930)
	Gail and Simon		0.1844	0.7975	607.4960	0.0181	192.5040	0.0335(0.1453)

The total sample size is set at n= 800, with $\pi = 0.5$ and $q_A \in \{0.1, 0.3, 0.6, 0.9\}$.

*EP: entire population;

*SP: subpopulation (efficacy/interaction) due to detection of the interaction with efficacy in subset k;

n_A and n_B are the mean sample size in each subset at the end of the study

Figure 1: Influence of the sample size reached at interim analysis on the proportion of decisions when $K = 2$ in Scenario 1, where there is no treatment effect and no treatment-by-subset interaction.

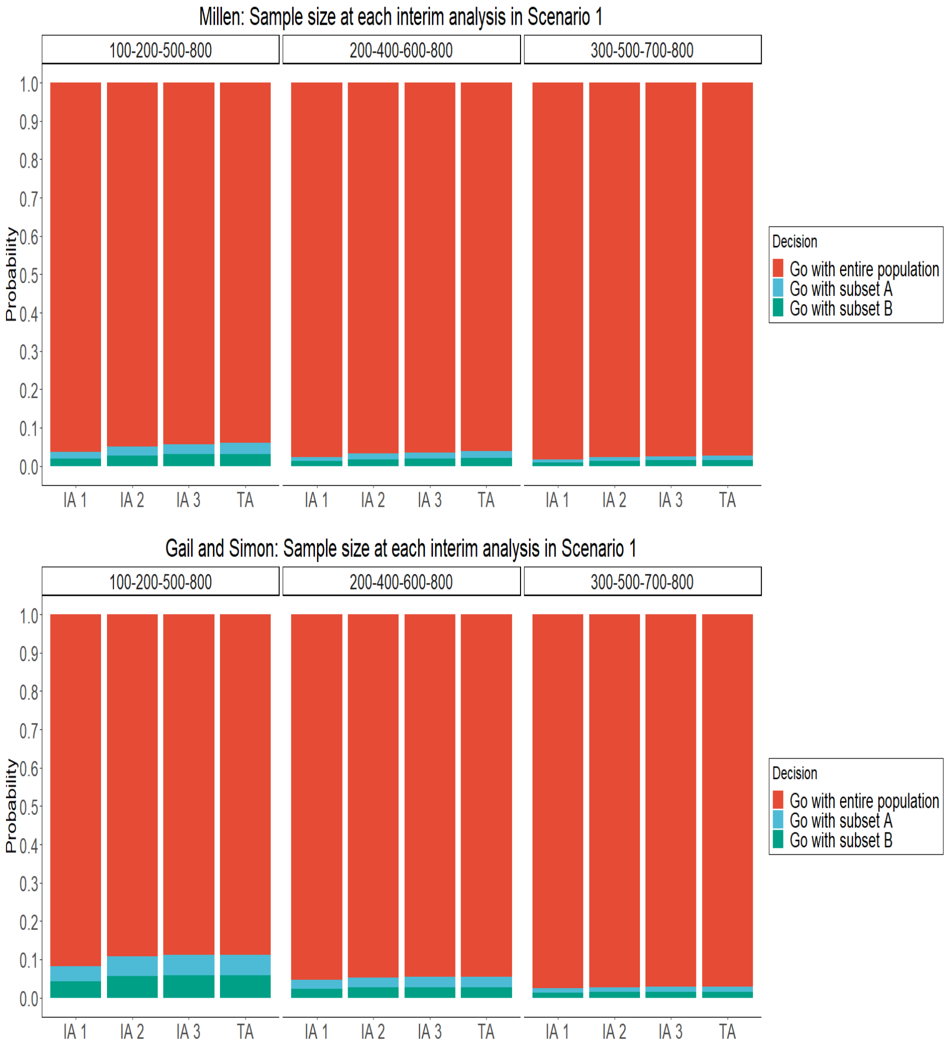

$n = 800$, $\pi = 0.5$ and $q_B = q_A = 0.5$
IA= Interim analysis, TA= Terminal analysis
